# Prognostic Impact of Tumor Budding in Intrahepatic Cholangiocellular Carcinoma

**DOI:** 10.7150/jca.63008

**Published:** 2022-05-06

**Authors:** Klara-Luisa Budau, Carlie S. Sigel, Luise Bergmann, Anne-Marie Lüchtenborg, Ulrich Wellner, Oliver Schilling, Martin Werner, Laura Tang, Peter Bronsert

**Affiliations:** 1 Institute of Surgical Pathology, Medical Center - University of Freiburg, Freiburg, Germany; 2 Faculty of Medicine, University of Freiburg, Germany; 3 Department of Pathology, Memorial Sloan-Kettering Cancer Center, New York, NY, USA; 4 German Cancer Consortium (DKTK) and Cancer Research Center (DKFZ), Heidelberg, Germany; 5 Tumorbank Comprehensive Cancer Center Freiburg, Medical Center - University of Freiburg, Germany; 6 Core Facility for Histopathology and Digital Pathology, Medical Center - University of Freiburg, Freiburg, Germany; 7 Clinic of Surgery, UKSH Campus Lübeck, Germany

**Keywords:** Intrahepatic cholangiocarcinoma, tumor buds, automated cell detection, independent prognosticator of survival

## Abstract

Background: Intrahepatic cholangiocarcinoma (ICC) represents an aggressive carcinoma with a dismal prognosis. For resection specimens, histopathological prognosticators are limited to standard AJCC parameters. Tumor budding (TB), a quantitative leviable parameter for tumor cell separation and infiltration is a promising prognostic factor for several cancers. This retrospective study investigated the prognostic impact of tumor budding in ICC, using a semi-automated approach.

Method: From the Memorial Sloan-Kettering Cancer Center pathology archives, tissue specimens from ICC patients were HE stained and digitized. Tumor budding was analyzed according to the International Tumor Budding Consensus Conference 2016 via QuPath in ten 0.785 mm² vision fields within the tumor center and the tumor-host interface. Within each field, automated QuPath cell detection was conducted and manually reviewed. Tumor budding was correlated with clinico-pathological parameters including AJCC 8^th^ edition classification, hepatitis status, age, ethnicity, treatment, sex, patient overall (OS) and recurrence free survival (RFS) via uni- and multivariate analyses.

Results: From 89 patients, 1780 Vision fields comprising 6006 tumor buds were analyzed and correlated with patients' OS and RFS. The median value for tumor budding in tumor budding hot spots was five within the tumor-host interface and six within the tumor center. Tumor budding correlated significantly with patient OS and RFS in uni- and multivariate analyses (p<0.001).

Conclusion: Our data supports tumor budding, assessed using a digitally enhanced technique, as an independent prognosticator in ICCs for patient's OS and RFS.

## Introduction

Intrahepatic cholangiocarcinoma (ICC) has a dismal five year patient's survival of up to 15% 1. ICCs are not accompanied by specific symptoms and consequently initial diagnosis is often made at an inoperable advanced-stage. Furthermore, only up to 35% of all patients are suitable for surgical intervention 2. For patients within an advanced tumor stage, the median overall survival is less than one year 3. There are diverse risk factors for ICC (primary sclerosing cholangitis, hepatolithiasis, hepatitis B / C virus infection; diabetes; obesity 4) and the molecular landscape is heterogeneous 4. However, a commonality of pathogenic ICC risk factors is chronic local inflammation.

Tumor budding, first described in 1949 by Imai et al. 5, is a quantifiable histologic pattern with proven prognostic value for several carcinomas and is applicable to grading schematics 6-8. To illustrate, the International Tumor Budding Consensus Conference (ITBCC) held in Bern in April 2016 proposed a scoring system for tumor buds for colorectal carcinomas. The ITBCC defined a tumor bud as a cell cluster detached from the main tumor mass comprising up to four tumor cells 9. This approach only considers tumor budding at the tumor-host interface and not at the tumor center. Since then, the ITBCC concept has also been applied to other cancers such as in hepatocellular 10, oral squamous cell 11 and bladder cancer 12.

Tumor size, multifocality, lymph node metastases, vascular invasion and tumor differentiation 13 are standard histopathological parameters for predicting ICC recurrence and overall survival, but there remains an opportunity for the discovery and validation of novel parameters such as tumor budding. Certain characteristics of ICC may make the quantification of tumor buds challenging, such as prominent desmoplastic stroma and an irregular interface with the adjacent hepatic parenchyma. Therefore, we developed a digitally enhanced approach for the marking and counting of tumor cell buds and subsequently tested for correlation between tumor budding and patients' overall (OS) and recurrence free survival (RFS) in a well characterized ICC cohort.

## Methods

From the Memorial Sloan-Kettering Cancer Center, New York, USA (MSKCC) pathology archives a cohort of 92 patients, with a primary surgical resection for ICC and a sufficient amount of tissue was identified. Thereof, three patients suffering from a combined hepatocellular-cholangiocellular carcinoma were excluded from the presented study. The resections took place at the Memorial Sloan-Kettering Cancer Center in the period between 2009 and 2018. All patients provided written informed consent for general clinical research. The study was conducted in accordance with the Declaration of Helsinki and approved by the local institutional review board (protocol number 16-1683A(3)).

All pathology slides and reports were reviewed by two pathologists at MSKCC. Histological diagnosis was assessed according to the World Health Organization classification 14, TNM classification has been assessed on the basis of the 8^th^ edition of American Joint Committee on Cancer (AJCC) 15.

One representative slide from each patient, including (if available) the tumor-host interface and the main tumor mass was selected and digitalized, using the VENTANA DP 200 slide scanner (software V1.0, routine focus method, 20-fold magnification, one focus layer).

### Clinico-pathological data

From the patient's records, the following data were retrieved: Age at *time* of operation (in years), sex (female, male), ethnicity (Caucasian, Black, Asian, and Hispanic), chronic hepatitis B or C virus infection, primarily sclerosing cholangitis and adjuvant radiotherapy. Histopathological data comprised Tumor size, T-classification, N- classification (in case of performed lymph node resection), AJCC 8^th^ edition stage, lymphangioinvasion, the presence of satellite nodule mass, liver capsule involvement and periductal infiltrating type.

The interval between resection and the date of disease recurrence comprised the recurrence free survival (RFS), which was defined as radiographic appearance of a new lesion (intrahepatic recurrence or metastasis) compatible with ICC or date of disease specific death for patients with no clinical date of recurrence. Patients without recurrence were censored at the date of last clinical encounter. Overall survival (OS) was defined as the interval between resection and death from any cause. Patients alive at last follow up, were censored at that date.

### Tumor budding

**Figure 1:**
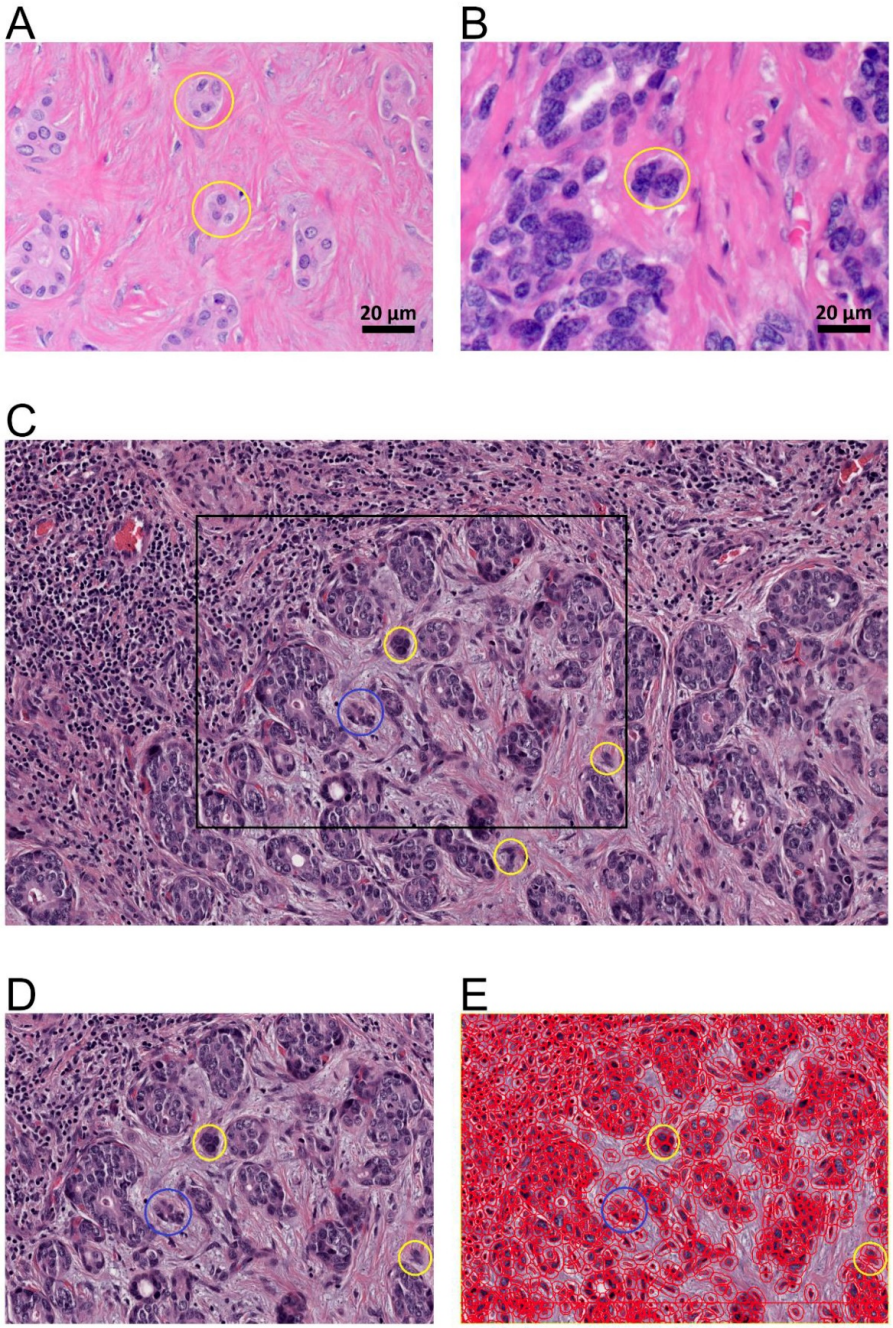
Tumor budding of intrahepatic cholangiocarcinoma. A., B. Tumor budding in HE stained ICC slides assessed with a light microscope at the tumor host interface to elucidate the morphology of the budding cells as a cell cluster (< five tumor cells) detached from the main tumor mass (yellow circles). C. Tumor budding in HE stained and digitalized ICC slides was assessed at the tumor host interface and within the tumor (intratumoral) demonstrating differently sized tumor buds. Tumor buds, demonstrated here in 10-fold magnification in QuPath, are defined as a cell cluster detached from the main tumor mass comprising less than five tumor cells (yellow circles). D. Rectangle from C in higher magnification (25-fold in QuPath) demonstrating tumor buds comprising of up to four cells (yellow circle) and cell group of more than 4 cells (blue circle) that are not detect as tumor bud. E. Evaluation of tumor buds using the automatic cell detection feature of QuPath enables evaluation in different magnifications, to detect and distinguish cells. Shown here at 25-fold magnification in QuPath.

Tumor budding (TB) was identified in one tissue slide on the basis of the recommendations of the International Tumor Budding Consensus Conference 2016 9. Tumor buds were defined as a single tumor cell or a cell cluster consisting of up to four tumor cells. They were analyzed at the tumor-host interface (peritumoral) and within the tumor center (intratumoral). Tumor bud assessment was performed in a vision field covering an area of 0.785 mm² via a digital assessed approach (see below) (Figure 1).

### Digital assessed approach

All images were analyzed in QuPath 16 (Version 0.1.2). To determine TB at the tumor-host interface and within the tumor center the recommendations for reporting tumor budding in colorectal cancer based on the International Tumor Budding Consensus Conference (ITBCC) 2016, which guided the digital assessed approach was followed. Qupath, an open source software for digital image analysis, allows analyses of digitalized high-resolution microscopic sections 16. QuPath enables the measurement and marking of areas, as well as of cells and cell groups. Although the applied QuPath version does not allow absolute automatic cell group detection in HE sections, the function “cell detection” facilitates the manual differentiation of tumor buds from larger tumor groups by detecting individual cells and only having to assess the number of cells.

Ten rectangles of H&E stained tissue slides were evaluated for both, the tumor-host interface and the tumor center. Each rectangle was standardized for an area of 0.785 mm^2^ as recommended. Therefore the number recorded on the microscope is divided by a normalization factor based on the eyepiece field number. Image pixel width and height were 0.465 µm each. In summary for each patient 20 rectangles with a 2150.54 Pixel (Px) width and 1688.17 Px height were determined via QuPath. Next, ten - peritumoral (including liver parenchyma) and intratumoral each - rectangles were randomly inserted. Rectangle overlaps were avoided when possible. If there was not enough space on peritumoral surfaces, as many rectangles as possible were placed. If no liver parenchyma was present, a total of 20 rectangles were placed intratumorally. Within the rectangles, all cells were detected and labelled with the QuPath application "Cell Detection" to facilitate differentiation.

Following the ITBCC guidelines, the amount of tumor buds in each rectangle was evaluated by using tenfold magnification to determine the rectangle with the highest tumor bud quantity, the TB hotspot. All TB hotspots were reevaluated by using a higher magnification (≥20-fold). TB hotspots were defined for both peritumoral and intratumoral areas. The TB hotspot with the highest tumor bud amount was included for the analyses.

### Tumor budding aggregation

According to ITBCC, TB was graded as low (zero to four tumor buds in one TB hotspot, per 0.785 mm^2^), intermediate (five to nine tumor buds in one TB hotspot) and high (≥ ten tumor buds in one TB hotspot). Intermediate and high TB was defined as TB positive, low TB as TB negative.

### Statistical Analysis

Statistical Analysis was performed using R (Version 3.6.0) and R Studio portable (Version 1.1.463). Descriptive statistics with median and percentage of total, as well as estimated median survival and estimated recurrence free survival were calculated (Table 1 and Table 2). The p-value for significance was defined ≤0.05, the p-value for statistical trends was set >0.05 and ≤0.15. For survival analysis and analysis of recurrence free survival Kaplan Meier method, Logrank-Test and univariate Cox Regression analysis were performed for age (dichotomized along the median patient age (<70 years) and old (≥ 70 years)), sex, ethnicity, hepatitis B/C-virus status, primarily sclerosing cholangitis-status, tumor size (dichotomized along the median tumor size (≥ 5.89 cm) and small (< 5.89 cm)), T- and N-Classification, AJCC 8^th^ edition stage 15, lymphangioinvasion, presence of satellite nodule mass, capsule involvement, periductal infiltrating type, and TB peritumoral, intratumoral and compiled grouped according to the recommendations of the ITBCC 9. Furthermore, included in survival analysis is adjuvant radiotherapy. No patient did receive any neoadjuvant therapy.

A multivariate Cox Regression analysis for overall and recurrence free survival was performed for T- and N-Classification, AJCC-stage, lymphangioinvasion, capsule involvement, periductal infiltrating type, adjuvant radiotherapy and TB peritumoral, intratumoral and compiled grouped according to the recommendations of the ITBCC 9. Subsequently, a nomogram was generated with the variables AJCC-stage, lymphangioinvasion, adjuvant therapy, and tumor budding compiled, which were significant in the multivariate analysis regarding overall survival.

Correlation between TB compiled and clinicopathological features, as well as correlations between TB peritumoral and TB intratumoral were calculated using Kendall-Rank-Correlation.

## Results

### Descriptive statistics: Patients characteristics

In total 89 patients (48 female (53.93%); 41 male (46.07%)) with a median age at time of surgical resection of 70 years (range 28 - 86 years) were included in this study. Ethnicity was distributed as follows: 74 (83.15%) Caucasian, four (4.49%) Black, nine (10.11%) Asian and one (1.12%) Hispanic. For one patient ethnicity was not reported. Of the 65 (73.03%) patients with available serologic testing results indicating chronic hepatitis B / C virus infection, eight (8.99%) were positive. Three (3.37%) patients had primary sclerosing cholangitis.

### Descriptive statistics: Histopathologic parameters

Median tumor size was 5.89 cm (range 1.80 - 13.5 cm) at the time of resection. According to 8^th^ AJCC classification 15 35 (39.33%) patients were classified as T1, 44 (49.44%) as T2, seven(7.87%) as T3 and three (3.37%) as T4. Local lymph node resection was performed for 58 (65.16%) patients, thereof 18 (20.22%) patients revealed local lymph node metastases (N+). According to AJCC staging 15 33 (37.08%) patients were classified as stage I, 29 (32.58%) as stage II and 27 (30.34%) as stage III.

Further histopathologic parameters included in this study were lymphangioinvasion (45 / 89 patients; 50.56%), satellite nodule mass (14/89 patients; 15.73%), liver capsule infiltration / involvement (9/89 patients; 10.11%) and periductal infiltration (8/89 patients; 8.99%). Furthermore 33 patients (37.08 %) were treated with adjuvant radiotherapy.

### Descriptive statistics: Tumor budding

For the 89 patients 6006 tumor buds were quantified, comprising both peritumoral and intratumoral tumor buds.

Peritumoral tumor budding was assessed in 80 (89.88%) cases at the tumor-host interface; in nine cases (10.11%) the tumor-host interface was not available. The mean tumor bud count in hotspots was 5.38, the median tumor bud count 5 (range 1 - 19 tumor buds). According to ITBCC^9^ 37 (41.57 %) ICCs were classified as TB low, 37 (41.57 %) ICCs as TB intermediate and 6 (6.74 %) ICCs as TB high.

Intratumoral tumor budding was evaluated in all 89 cases. The mean intratumoral tumor bud count was 8.52 and the median six (range 2 - 120) per hotspot. According to ITBCC 9 31 (34.83 %) ICCs were classified as TB low, 35 (39.33 %) ICCs as TB intermediate and 23 (25.84 %) ICCs as TB high.

To analyse the intratumoral comparability of the ITBCC classification (TB- low, medium, high) within the peri- and intratumoral region, 80 cases were compared. Of the 80 examined cases both peritumoral and intratumoral for tumor budding, concordance was observed in 52 (62%) cases. In 25 (31.25%) cases TB low was detected peritumoral and intratumoral, in 21 (26.25%) cases equally TB intermediate and in six (7.5%) cases TB high. Only in one (1.25%) case TB peritumoral low and TB intratumoral high was detected. In four (5%) cases, TB peritumoral was assessed as intermediate but intratumoral as low. No case was found with TB peritumoral high and only intermediate or low TB intratumoral. The group of cases with TB peritumoral low and TB intratumoral intermediate comprised 11 (13.75%) patients. Furthermore, the group with TB peritumoral intermediate and TB intratumoral high included 12 (15%) cases. As the data for TB peritumoral and TB intratumoral are not normally distributed, the correlation was tested using the Kendall rank method. In this, the two show a significant correlation (p<0.001) (Table 6).

For each ICC, TB at the tumor-host interface and intratumoral were compared and the highest amount of tumor buds was chosen for the final TB grading. This represents the compiled tumor bud count. The total mean compiled tumor bud count was 8.88, the median seven (range 2 - 120). According to ITBCC 9 26 (29.21 %) ICCs were classified as TB low, 38 (42.70 %) ICCs as TB intermediate and 25 (28.09 %) ICCs as TB high.

### Analysis via Kaplan Meier method and Logrank-Test

The estimated patient mean overall survival was 36.33 months, the patient median overall survival was 27.50 months (range 0 - 115 months). At last follow-up 45 (50.56%) patients were deceased. The estimated patient mean recurrence free survival was 22.54 months, the patient median recurrence free survival 13 months (range 0 - 102 months). At the last follow-up 62 (69.66%) patients suffered from an ICC recurrence.

Analyzing all clinico-pathological parameters via Kaplan Meier method and Logrank-Test for patients' overall survival, prognostic significance for overall survival was estimated for T-classification (p=0.017), N-classification (p<0.001), AJCC 8^th^ edition stage^15^ (p<0.001), lymph vessel infiltration (p<0.001), liver capsule infiltration / involvement (p=0.024), periductal infiltrating type (p=0.038), adjuvant radiotherapy (p=0.022), TB peritumoral (p<0.001), intratumoral (p<0.001) and compiled (p<0.001) (Table 1).

Analyzing all clinico-pathological parameters via Kaplan Meier method and Logrank-Test for patients' recurrence free survival, Kaplan Meier method revealed statistical significances for N-Classification (p<0.001), AJCC 8^th^ edition stage^15^ (p<0.001), lymphangioinvasion (p= 0.01), adjuvant radiotherapy (p= 0.01), for TB peritumoral (p< 0.001), intratumoral (p< 0.001) and for compiled (p= 0.003) (Table 2).

**Figure 2:**
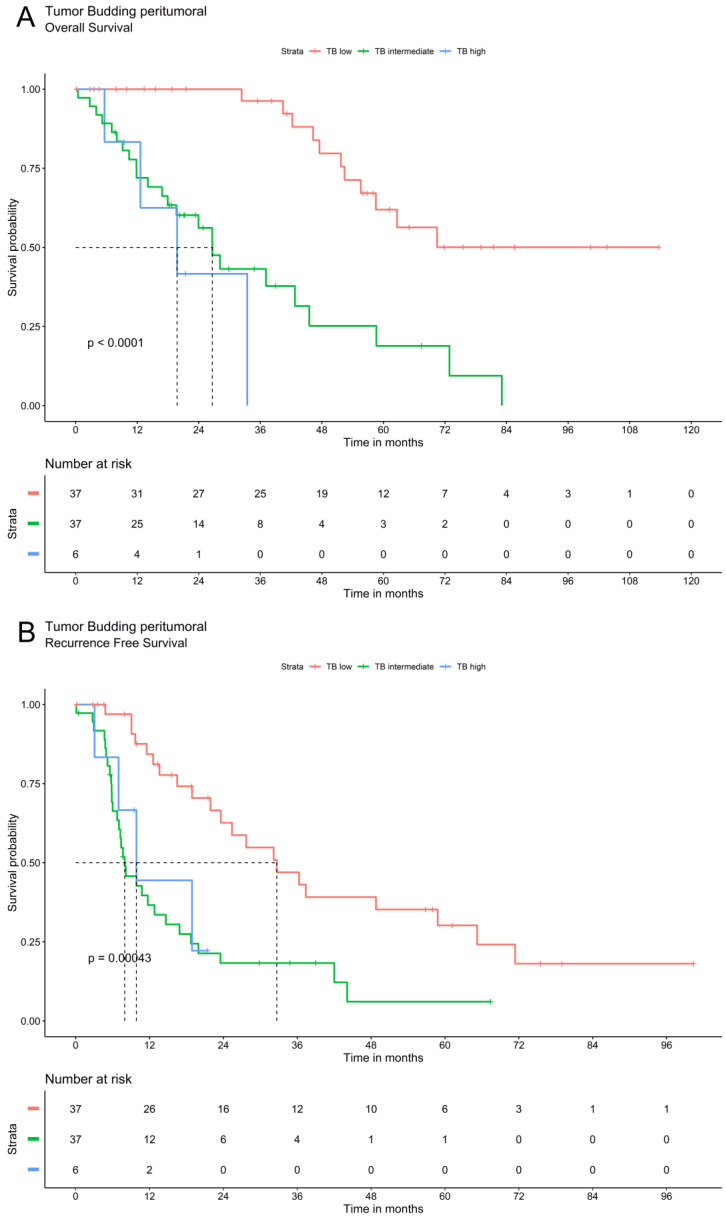

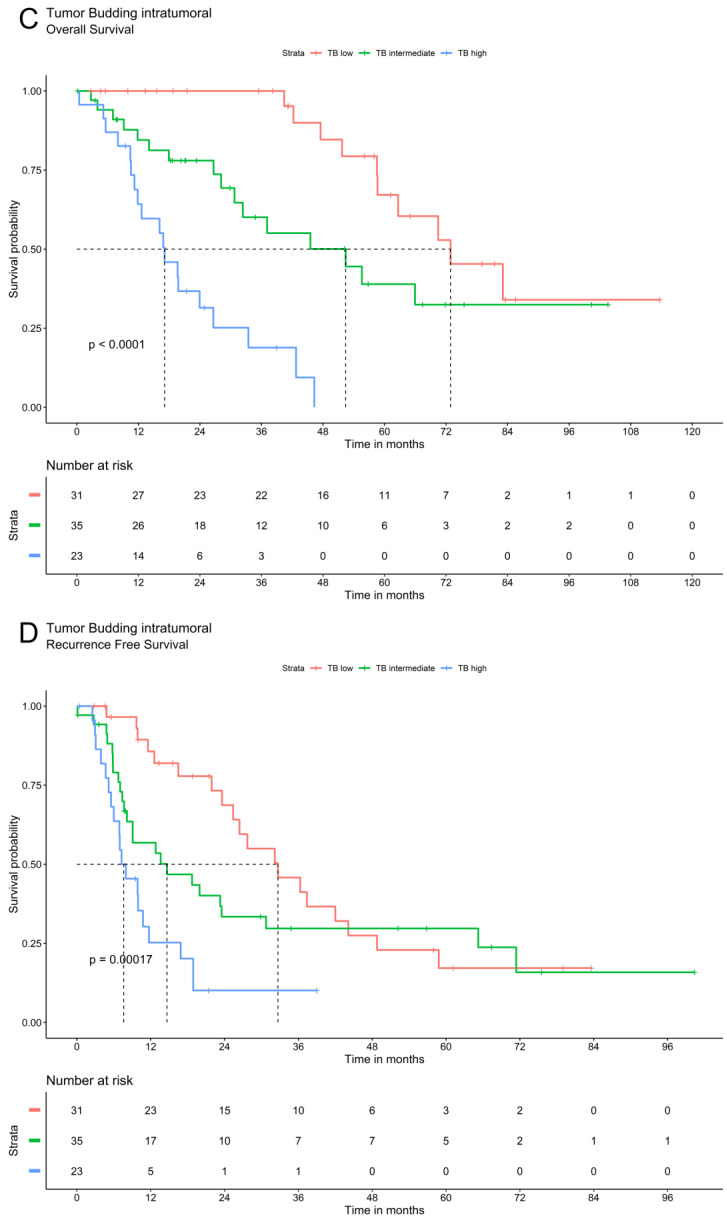

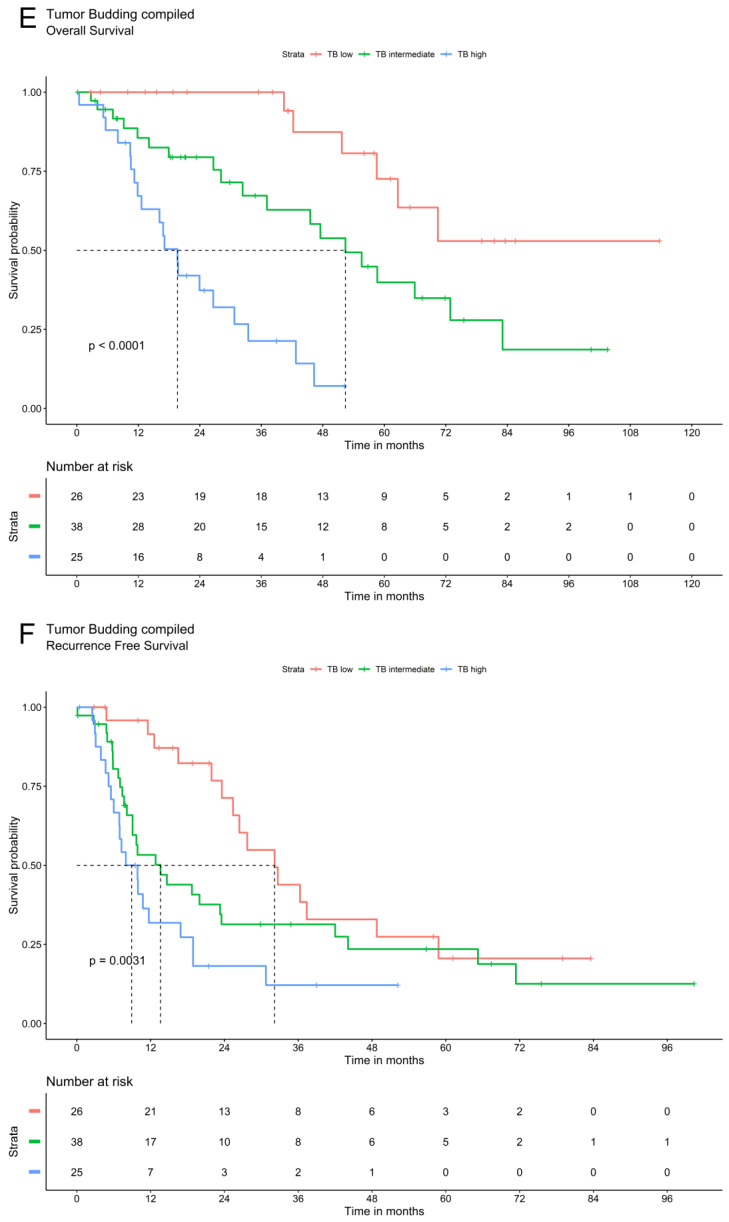
Survival analysis of ICC demonstrate that tumor budding correlates significantly with a worse OS and RFS. A. OS of Tumor budding peritumoral low, intermediate and high according to ITBCC. B. RFS of Tumor budding peritumoral low, intermediate and high according to ITBCC. C. OS of Tumor budding intratumoral low, intermediate and high according to ITBCC. D. RFS of Tumor budding intratumoral low, intermediate and high according to ITBCC. E. OS of Tumor budding (compiled) low, intermediate and high according to ITBCC. F. RFS of Tumor budding (compiled) low, intermediate and high according to ITBCC

Tumor budding was analyzed via Kaplan Meier method using a three-tier grading system. The three patients' groups (TB high, TB intermediate and TB low) showed significant differences in overall and recurrence free survival for TB in peritumoral or intratumoral area as well as for compiled data. Patients with low TB had the most favorable recurrence survival whereas high TB was associated with the most unfavorable outcomes. In patients with intermediate TB survival ranged between these two extremes. The overall and recurrence free survival declines significantly with a higher grade of TB (Figure 2).

### Univariate and multivariate survival analysis

In univariate analysis prognostic significance was shown by T-Classification (HR=1.59; p=0.01), AJCC 8^th^ edition stage^15^ (HR=2.64; p< 0.001), lymphangioinvasion (HR=2.97; p=0.001), capsule involvement (HR=2.86; p<0.03), adjuvant radiotherapy (HR=1.99; p=0.02) and TB peritumoral (HR=3.65; p<0.001), intratumoral (HR=3.49; p<0.001) and compiled (HR=3.60; p<0.001).

A stepwise multivariate Cox regression model with T- and N-Classification, AJCC 8^th^ edition stage 15, lymphangioinvasion, capsule involvement, periductal infiltrating type, adjuvant radiotherapy and TB peritumoral, intratumoral and compiled was performed.

**Figure 3:**
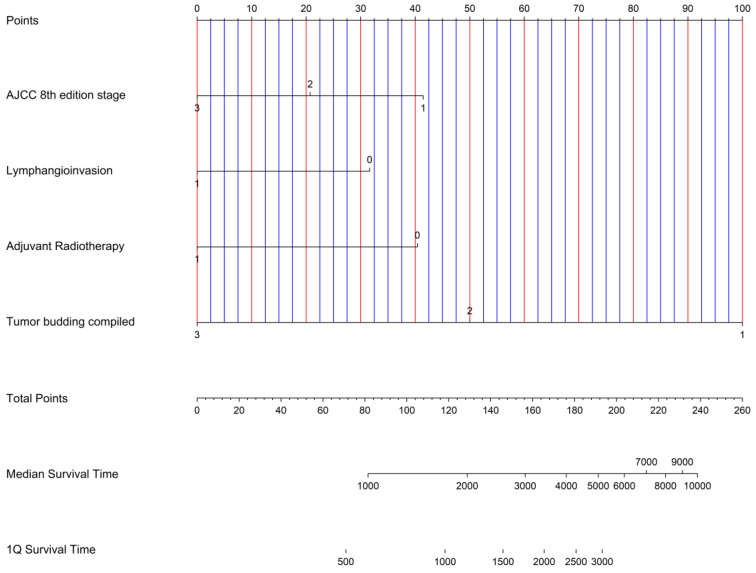
Nomogram for possible risk stratification of ICC knowing AJCC 8 th edition stage, lymphangioinvasion, adjuvant radiotherapy and Tumor budding compiled.

AJCC 8^th^ edition stage (HR=2.70; p<0.01), lymphangioinvasion (HR=2.61; p=0.03), adjuvant radiotherapy (HR=2.21; p=0.04) and TB peritumoral (HR=3.30; p< 0.001), intratumoral (HR=3.54; p<0.001) and compiled (HR=3.28; p<0.001) demonstrated a statistical independent prognostic significance for overall survival (Table 3). A graphical representation of the multivariate analysis by means of a nomogram has been made (Figure 3).

Univariate and multivariate Cox regression analysis were performed for recurrence free progression as well. In univariate analysis prognostic significance was shown by T-Classification (HR=1.38; p=0.04), AJCC 8^th^ edition stage (HR=2.84; p< 0.001), lymphangioinvasion (HR=0.50; p< 0.01), adjuvant radiotherapy (HR=0.49; p=0.01) and TB peritumoral (HR=2.06; p< 0.001), intratumoral (HR= 1.92; p<0.001) and compiled (HR=1.77; p<0.001) (Table 4).

Multivariate analysis comprising T- and N-Classification, AJCC 8^th^ edition stage^15^, lymphangioinvasion, capsule involvement, periductal infiltrating type, adjuvant radiotherapy and peritumoral, intratumoral and compiled TB revealed independent significant correlations for adjuvant radiotherapy (HR=2.49; p<0.01) and TB peritumoral (HR=2.27; p< 0.001), intratumoral (HR=1.94; p< 0.001) and compiled (HR=1.72; p< 0.01) (Table 4).

### Kendall-Rank-Correlation analysis

Shapiro-Wilk-Test revealed no normal distribution for tumor budding. For this reason, Kendall-Rank-Correlation was performed to assess possible correlation between tumor budding and clinico-pathological features.

A statistical trend could be identified for advanced AJCC 8^th^ edition stage 15 (p=0.070) (Table 5).

## Discussion

Using a digital enhanced approach, the present study shows the prognostic impact of tumor budding in ICC on overall survival (OS) and recurrence free survival (RFS) including clinico-pathological features.

As a quantifiable histologic pattern with prognostic value for various carcinomas, tumor budding was first described by Imai et al in 1949 17. Probably due to reduced cell adhesion, this morphological phenomenon can be observed in the advanced stage of neoplasia 18. Isolated tumor cells or small groups of tumor cells, understood as tumor buds, consequently detach from the main tumor mass and the neoplastic epithelium and migrate a short distance into the surrounding tissue / the neoplastic stroma 19,20. Tumor cells singulation, respectively increased tumor budding, directly correlates with a poorer tumor cell differentiation, an elevated tumor stage and subsequently poorer patient overall survival 21,22. Simplified, tumor budding can be understood as histomorphological representation of the cell's migratory and invasive properties.

We evaluated TB peritumoral at the tumor-host interface, intratumoral in the tumor mass center and the compiled data. From 89 patients 1780 vision fields comprising 6006 tumor buds were analyzed. Using a three-tier grading system intermediate and high tumor budding was present in 70.79 % (63/89) of all patients. Patients with low TB have a favorable outcome compared with patients with high and intermediate TB (overall and recurrence free survival). We demonstrate that OS and RFS declines significantly with a higher grade of TB. Furthermore, TB correlated significantly with patient OS and RFS in uni- and multivariate analyses (p<0.001) and with patient nodal status in bivariate analyses. In addition, a correlation between tumor budding peritumoral and intratumoral was observed.

A nomogram was created based on our results in multivariate analysis (Figure 3). It determines the statistical median survival of ICC patients considering AJCC stage, lymphangioinvasion, adjuvant radiotherapy and TB compiled. In perspective, our results can be validated by further preferably prospective studies based on the nomogram.

To our knowledge this study is the first reporting tumor budding in ICC using digital enhanced approach.

We adapted the recommendations of ITBCC for reporting TB on a microscope for a digital approach via QuPath, an open-source, user-friendly software for digital image analysis. Due to its ability to work with high resolution digital scans of microscopic slides it represents an attractive platform for biomedical research 16. We designed a semi-automated approach that offered the possibility to detect and permanently mark tumor buds. Automated cell detection also facilitated distinguishing tumor buds from larger tumor cell groups. Compared to “analog” microscopy, using QuPath enables the reevaluation of tumor buds at any time. These features offer a high degree of reproducibility and comparability which is advantageous for the research and clinical diagnostic settings.

As there is no standardized method for reporting tumor budding, various applications to count tumor buds exist. The definition of tumor buds as tumor cells or clusters of cells with a distinct morphology as a characteristic of tumor dedifferentiation 20,23,24 goes back to Gabbert et al. 24 and Hase et al. 25. Since then, the approaches in literature vary markedly. Different definitions concerning the size of tumor buds, e.g. whether groups of up to four or up to five tumor cells should be counted, exist 9,19,26,27. Additionally the staining approach for analyzing tumor budding (hematoxylin / eosin versus immunohistochemistry) 20,26 and the assessment via hotspot method in an area of 0.785 mm² or via mean tumor bud count in 40-fold magnification high power-fields have been discussed 28.

For this study, we adopted the recommendations for reporting tumor budding in colorectal cancer based on the International Tumor Budding Consensus Conference (ITBCC) 9.

ITBCC determined eleven statements, including the definition of a tumor bud as a single tumor cell or a cell cluster of up to four tumor cells and the assessment of TB in a hotspot field measuring 0.785 mm² by using an H&E slide. Furthermore the participants of ITBCC suggested a three-tier grading system in order to access more granular risk stratification 9. Tanaka et al. 29 graded according to ITBCC in low, intermediate and high, but combined intermediate and high for statistical analysis to tumor budding positive cases. In consequence only tumor budding positive and tumor budding negative cases were distinguished. In contrast, our data suggests that a three-tier grading system also identifies a group with intermediate risk that may benefit from special risk stratification and specially adapted therapy in future clinical trials.

Of note, TB has mainly been considered a phenomenon at the tumor-host interface. Tumor budding happening in the main tumor mass was only little considered until the ITBCC.

The ITBCC participants raised the possibility of the existence of intratumoral tumor buds given the associations with lymph node metastasis in colorectal carcinoma 9. Nevertheless the evidence for intratumoral tumor budding is still weak 30,31. Intratumoral tumor budding describes detached single tumor cells or clusters within the tumor but without contact to the tumor bulk. Few studies have discussed this phenomenon using the same criteria as for peritumoral tumor budding 31,32. High intratumoral tumor budding and tumor differentiation correlate with advanced T- and N-Classification and lymphatic invasion in colorectal carcinoma and suggest a comparable significance for intratumoral and peritumoral tumor budding 32,33. Our data suggest that intratumoral tumor budding is as important in ICC as it is in colorectal carcinoma 9,32. Overall, our data strengthens the view of the importance of intratumoral tumor budding in cancer and supports the data of Lugli et al. 32.

Furthermore, our data suggest that in ICC tumor budding is significant regardless of whether it is intra- or peritumoral. This is fortunate, because needle core liver biopsies often lack orientation with the tumor-liver interface yet remain a viable option for additional studies evaluating the predictive value of this histologic feature in pre-operative or non-operative setting.

Our results are in line with the recently published data from Tanaka et al. 29 and support the suggestion of tumor budding as a relevant prognostic factor in histopathologic evaluation for ICC.

Due to late clinical presentation, ICC has a dismal prognosis and a curative resection is often not possible 34. Several other studies have discussed the lack of prognostic stratification of patients with ICC 35 and emphasize the need for new prognostic and predictive factors for this carcinoma to adapt current therapeutic regimes.

Tumor budding has already been investigated as a comprehensive and independent prognostic factor in various malignant tumors e.g., colorectal carcinoma 30-32,36,37, pancreatic ductal adenocarcinoma 27,28, extrahepatic cholangiocarcinoma 38,39, gallbladder cancer 39, breast cancer 8,40 and oral squamous cell cancer 41. Given its easy analysis irrespective of localization within the tumor mass should be prospectively integrated into reemerging WHO classifications for ICC.

## Conclusions

Tumor budding, regardless of whether intra- or peritumoral, is a significant prognosticator for ICCs since it is strongly associated with an advanced tumor stage and subsequently with a dismal prognosis for patients' disease-free progression and overall survival. This histologic feature can be easily accessed using digital imaging platform we described. Together with the data from Tanaka et al. 29, our data emphasize that tumor budding is a relevant histopathological parameter in ICCs.

We advocate for a consensus on a standardized method for tumor budding assessment in ICC and consideration of this feature for standard reporting.

## Figures and Tables

**Table 1:** Clinico-Pathological features of ICC using Kaplan-Meier-method for Overall Survival (OS).

Parameter	N	of total	in %	estimated median survival (months)	Range (months)	P
**Overall Survival**	Mean 36.33 months	27.50	0 to 115			
dead at last follow up	45	0.51	50.56			
**Recurrence free Survival**	Mean 22.54 months	13	0 to 102			
Relaps	62	0.70	69.66			
**Age at Resection**	Mean 67 years	69	28 to 86			
<70	49	0.55	55.06	47.07	33.43 to NA	
≥70	40	0.45	44.94	57.43	34.57 to 85.83	0.98
**Sex**						
Female	48	0.54	53.93	49.10	43.63 to 75.30	
Male	41	0.46	46.07	57.43	27.50 to NA	0.69
**Ethnicity**						
Caucasian	74	0.83	83.15	53.43	38.33 to 72.80	
Black	4	0.04	4.49	29.07	4.13 to NA	
Asian	9	0.10	10.11	47.83	33.43 to NA	
Hispanic	1	0.01	1.12	NA	NA	
Not reported	1	0.01	1.12	NA	NA	0.97
**chronic Hepatitis B/C-virus infection**						
Yes	8	0.09	8.99	54.17	33.43 to NA	
No	57	0.64	64.04	47.83	41.77 to 85.83	
Not tested	24	0.27	26.97	49.10	27.50 to NA	0.96
**Clinical PSC**						
Yes	3	0.03	3.37	41.77	29.07 to NA	
No	86	0.97	96.63	54.17	43.63 to 74	0.28
**Tumorsize**	Mean: 5.89 cm	5.60	1.80 to 13.50			
< 5.89 cm	48	0.54	53.93	60.57	38.33 to NA	
≥ 5.89 cm	41	0.46	46.07	47.83	27.50 to 68.13	0.13
**T-Classification**						
T1	35	0.39	39.33	72.80	54.17 to NA	
T2	44	0.49	49.44	38.33	29.07 to 68.13	
T3	7	0.08	7.87	27.53	10.77 to NA	
T4	3	0.03	3.37	727	5.80 to NA	0.017*
**N-Classification**						
N+	18	0.20	20.22	16.67	12.27 to 38.33	
N0	40	0.45	44.94	75.30	49.10 to NA	
No lymph nodes resected	31	0.35	34.83	60.57	47.07 to NA	<0.001*
**AJCC 8th edition stage**						
I	33	0.37	37.08	72.80	57.43 to NA	
II	29	0.33	32.58	60.47	47.07 to NA	
III	27	0.30	30.34	17.40	12.27 to 38.33	<0.001*
**Lymphangioinvasion (L)**						
**L+**	**45**	**0.51**	**50.56**	**33.43**	**18.53 to 53.43**	
L0	44	0.49	49.44	72.80	54.17 to NA	<0.001*
**Satellite Nodule Mass**						
Yes	14	0.16	15.73	29.07	8.27 to NA	
No	75	0.84	84.27	53.43	43.63 to 72.80	0.16
**Capsule Involvement**						
Yes	9	0.10	10.11	27.53	8.27 to NA	
No	80	0.90	89.89	54.17	43.63 to 75.30	0.024*
**Periductal infiltrating type**						
Yes	8	0.09	8.99	27.50	8.27 to NA	
No	62	0.70	69.66	57.43	44.17 to NA	0.038*
Not reported	19	0.21	21.35	34.57	14.53 to NA	0.05
**Adjuvant Radiotherapy**						
Yes	33	0.37	37.08	27.57	1.74 to 60.47	
No	56	0.63	62.92	64.73	44.17 to NA	0.022*
Tumor budding peritumoral*						
low (0-4/0.785mm²)	37	0.42	41.57	NA	58.50 to NA	
intermediate (5-9/0.785mm²)	37	0.42	41.57	26.60	17.90 to 58.60	
high (≥10/0.785mm²)	6	0.07	6.74	19.80	12.60 to NA	<0.001*
**Tumor budding intratumoral**						
low (0-4/0.785mm²)	31	0.35	34.83	72.90	58.60 to NA	
Intermediate (5-9/0.785mm²)	35	0.39	39.33	52.40	30.70 to NA	
high (≥10/0.785mm²)	23	0.26	25.84	17.10	11.90 to 33.50	<0.001*
**Tumor budding compiled**						
low (0-4/0.785mm²)	26	0.29	29.21	NA	62.60 to NA	
Intermediate (5-9/0.785mm²)	38	0.43	42.70	52.40	37.10 to NA	
High (≥10/0.785mm²)	25	0.28	28.09	19.60	12.60 to 33.60	<0.001*
NA= Not available *P<0.05.						

* Tumor budding peritumoral was compiled at the tumor host interface. In 9/89 patients, corresponding tissue samples were not available. Therefore, the number of patients at Tumor budding peritumoral differs from the other listed clinico-pathological parameters.

**Table 2:** Clinico-pathological features of ICC using Kaplan-Meier-method for Recurrence free Survival (RFS). Intratumoral

Parameter	N	of total	in %	estimated median recurrence free Survival (months)	Range (months)	P
**Age at Resection**		Mean 67 years		69	28 to 86	
<70	49	0.55	55.06	17.03	10.27 to 26.23	
≥70	40	0.45	44.94	24.07	12.10 to 38.63	0.44
**Sex**						
Female	48	0.54	53.93	19.53	11.10 to 33.23	
Male	41	0.46	46.07	13.23	9.37 to 37.50	0.56
**Ethnicity**						
Caucasian	74	0.83	83.15	19.33	11.10 to 31.77	
Black	4	0.04	4.49	15.13	0.10 to NA	
Asian	9	0.10	10.11	19.53	9.37 to NA	
Hispanic	1	0.01	1.12	NA	NA	
Not reported	1	0.01	1.12	NA	NA	0.74
**chronic Hepatitis B/C-virus infection**						
Yes	8	0.09	8.99	14.07	11.93 to NA	
No	57	0.64	64.04	17.03	10.27 to 33.23	
Not tested	24	0.27	26.97	19.33	10 to NA	0.90
**Clinical PSC**						
Yes	3	0.03	3.37	15.13	13 to NA	
No	86	0.97	96.63	19.53	11.10 to 28.63	0.37
**Tumorsize**		Mean: 5.89 cm		5.60	1.80 to 13.50	
< 5.89 cm	48	0.54	53.93	24.30	15.13 to 38.63	
≥ 5.89 cm	41	0.46	46.07	10.20	7.93 to 27.30	0.74
**T-Classification**						
T1	35	0.39	39.33	20.60	13 to NA	
T2	44	0.49	49.44	17.03	9.37 to 31.77	
T3	7	0.08	7.87	10	8.23 to NA	
T4	3	0.03	3.37	7.27	3.17 to NA	0.23
**N-Classification**						
N+	18	0.20	20.22	8.23	5.73 to 24.30	
N0	40	0.45	44.94	20.60	17.43 to 50.40	
No lymph nodes resected	31	0.35	34.83	17.03	11.93 to NA	<0.001*
**AJCC 8th edition stage**						
I	33	0.37	37.08	24.37	14.07 to NA	
II	29	0.33	32.58	24.07	15.13 to 50.40	
III	27	0.30	30.34	8.23	6.20 to 24.30	<0.001*
**Lymphangioinvasion (L)**						
L+	45	0.51	50.56	12.120	8.23 to 24.30	
L0	44	0.49	49.44	24.37	14.07 to NA	0.01*
**Satellite Nodule Mass**						
Yes	14	0.16	15.73	13.23	5.33 to NA	
No	75	0.84	84.27	20.60	12.10 to 33.23	0.16
**Capsule Involvement**						
Yes	9	0.10	10.11	8.40	8.23 to NA	
No	80	0.90	89.89	19.53	13 to 28.63	0.27
**Periductal infiltrating type**						
Yes	8	0.09	8.99	17.03	6.20 to NA	
No	62	0.70	69.66	19.53	12.10 to 38.63	
Not reported	19	0.21	21.35	19.53	7.63 to NA	0.25
**Adjuvant Radiotherapy**						
Yes	33	0.37	37.08	9.37	7.63 to 20.60	
No	56	0.63	62.92	24.30	17.43 to 38.63	0.01*
Tumor budding peritumoral*						
**low** (0-4/0.785mm²)	37	0.42	41.57	32.68	23.58 to 71.40	
**intermediate** (5-9/0.785mm²)	37	0.42	41.57	7.97	6.74 to 16.90	
**high** (≥10/0.785mm²)	6	0.07	6.74	9.87	6.97 to NA	<0.001*
**Tumor budding intratumoral**						
**low** (0-4/0.785mm²)	31	0.35	34.83	32.68	25.39 to 48.80	
**Intermediate** (5-9/0.785mm²)	35	0.39	39.33	14.65	8.13 to 65.20	
**high** (≥10/0.785mm²)	23	0.26	25.84	7.61	6 to 16.90	<0.001*
**Tumor budding compiled**						
**low** (0-4/0.785mm²)	26	0.29	29.21	32.16	25.39 to NA	
**Intermediate** (5-9/0.785mm²)	38	0.43	42.70	13.61	9.06 to 42	
**High** (≥10/0.785mm²)	25	0.28	28.09	8.92	6.90 to 19	0.003*
NA = Not available *P<0.05						

* Tumor budding peritumoral was compiled at the tumor host interface. In 9/89 patients, corresponding tissue samples were not available. Therefore, the number of patients at Tumor budding peritumoral differs from the other listed clinico-pathological parameters.

**Table 3:** Univariate and multivariate survival analysis for Overall Survival (OS)

	Univariate Cox regression			~OS	Multivariate Cox Regression			~OS
Parameter	HR	95% CI		P	HR	95% CI		P
**Age at Resection**	1.01	0.99 to 1.04		0.34	n.i.			
<70	0.99	0.55 to 1.79		0.98				
≥70								
**Sex**	1.13	0.63 to 2.04		0.69	n.i.			
Female								
Male								
**Ethnicity**					n.i.			
Caucasian	0.91	0.36 to 2.33	0.85					
Black	1	0 to 6.51	0.79					
Asian								
Hispanic	0.00	0.00	NA	1.00				
Not Reported	0.00	0.00	NA	1.00				
**chronic Hepatis B/C-virus infection**					n.i.			
Yes	0.33	2.70 to 0.92		0.94				
No								
Not Reported	0.55	2.13 to 0.81						
**Clinical PSC**	0.58	6.25 to 0.28		0.28	n.i.			
**Tumorsize**	0.63	0.35 to 1.15		0.13	n.i.			
<5.89 cm								
≥ 5.89 cm								
**T-Classification**	1.59	1.14 to 2.23		0.01*	0.48	0.22 to 1.04		0.06
**N-Classification**	1.00	0.99 to 1.00		0.75	1.00	0.99 to 1.00		0.26
**AJCC 8th edition stage**	2.64	1.75 to 3.99		<0.001*	2.70	1.39 to 5.25		<0.01*
**Lymphangioinvasion**	2.97	1.55 to 5.68		0.001*	2.61	1.12 to6.05		0.03*
**Satellite Nodule Mass**	1.79	0.79 to 4.04		0.16	n.i.			
**Capsule Involvement**	2.86	1.10 to 7.44		0.03*	2.64	0.70 to 9.99		0.15
**Periductal infiltrating type**	1.00	0.99 to 1.00		0.18	1.00	0.99 to 1.00		0.17
**adjuvant Radiotherapy**	1.99	1.09 to 3.63		0.02*	2.21	1.02 to 4.80		0.04*
**Tumor budding peritumoral**	3.65	2.18 to 6.13		<0.001*	3.30	1.82 to 6.02		<0.001*
**Tumor budding intratumoral**	3.49	2.19 to 5.54		<0.001*	3.54	2.15 to 5.83		<0.001*
**Tumor budding compiled**	3.60	2.21 to 5.89		<0.001*	3.28	1.94 to 5.52		<0.001*
n.i. = not included *P<0.05								

**Table 4:** Univariate and multivariate survival analysis for Recurrence free Survival (RFS)

	Univariate Cox regression			~RFS	Multivariate Cox regression			~RFS
Parameter	HR		95% CI	P				P
**Age at Resection**	1.00	0,9779 to 1,023		0.98	n.i.			
<70	1.22	0.73 to 2.03		0.44				
≥70								
**Sex**	1.16	0.70 to 1.92		0.56	n.i.			
Female								
Male								
**Ethnicity**					n.i.			
White	0.75	0.35 to 1.59	0.46					
Black	1.25	0.33 to 4.75	0.74					
Asian								
Hispanic	0.00	0.00	NA	1.00				
Not Reported	0.00	0.00	NA	1.00				
**chronic Hep B/C-virus infection**				n.i.				
Yes	0.90	0.38 to 2.11	0.80					
No								
Not Reported	0.88	0.48 to 1.59	0.67					
**Clinical_PSC**	0.59	0.18 to 1.91	0.37	n.i.				
**Tumorsize**	1.10	1,01 to 1,20		0.03	n.i.			
<5.89 cm	0.74	0.45 to 1.22		0.23				
≥5.89 cm								
**T-Classification**	1.38	1,02 to 1,87	0.04*	0.67	0.35 to 1.27	0.21		
**N-Classification**	0.40	0.20 to 5.35	<0.01*	1.00	0.99 to 1.00	0.27		
**AJCC 8th edition stage**	2.84	1.51 to 5.35	<0.01*	1.78	0.96 to 3.28	0.07		
**Lymphangioinvasion**	0.50	0.30 to 0.84		<0.01*	1.77	0.91 to 3.44		0.09
**Satellite Nodule Mass**	0.63	0.33 to 1.21		0.17	n.i.			
**Capsule Involvement**	0.60	0.24 to 1.52		0.28	1.25	0.43 to 3.59		0.68
**Periductal infiltrating type**	1.80	0.80 to 1.01		0.15	1.00	0.99 to 1.00		0.74
**adjuvant Radiotherapy**	0.49	0.29 to 0.81		0.01*	2.49	1.41 to 4.38		<0.01*
**Tumor budding peritumoral**	2.06	1.369 to 3.09		<0.001*	2.27	1.45 to 3.56		<0.001*
**Tumor budding intratumoral**	1.92	1.356 to 2.73		<0.001*	1.94	1.34 to 2.81		<0.001*
**Tumor budding compiled**	1.77	1.26 to 2.49		<0.001*	1.72	1.20 to 2.45		<0.01*
n.i. = not included *P<0.05								

**Table 5:** Kendall-Rank-Correlation between Tumor budding compiled and Clinicopathologic Features

Parameter	Kendall τ	P
**Age at Resection**	-0.03	0.727
**Sex**	-0.07	0.493
**Ethnicity**	-0.14	0.147
**chronic Hep B/C-virus infection**	-0.01	0.906
**Clinical PSC**	-0.15	0.129
**Tumorsize**	-0.05	0.559
**T-Classification**	0.09	0.378
**N-Classification**	0.08	0.417
**AJCC 8th edition stage**	0.17	0.070
**Lymphangioinvasion**	0.13	0.210
**Satellite Nodule Mass**	0.01	0.952
**Capsule Involvement**	0.10	0.330
**Periductal infiltrating type**	-0.05	0.649
**adjuvant Radiotherapy**	0.07	0.493

**Table 6:** Concordance between Tumor budding peritumoral and intratumoral

		*Tumor budding intratumoral*			
***Tumor budding peritumoral***		**low**	**intermediate**	**high**	
	**low**	25	11	1	37
	**intermediate**	4	21	12	37
	**high**	0	0	6	6
		29	32	19	80
***Kendall-Rank-Correlation between Tumor budding peritumoral and intratumoral***		Kendall τ		P	
		0.64		<0.001	

## Abbreviations

ICC: Intrahepatic cholangiocarcinoma
TB: Tumor budding
OS: patient overall survival
RFS: recurrence free survival
ITBCC: International Tumor Budding Consensus Conference
